# Smartphone Use and Psychological Well-Being Among College Students in China: A Qualitative Assessment

**DOI:** 10.3389/fpsyg.2021.708970

**Published:** 2021-09-09

**Authors:** Cheng Dai, Zixue Tai, Shan Ni

**Affiliations:** ^1^School of Journalism and Communication, Minjiang University, Fuzhou, China; ^2^School of Journalism and Media, University of Kentucky, Lexington, KY, United States; ^3^School of Economics and Management, Minjiang University, Fuzhou, China

**Keywords:** smartphone use disorder, smartphone dependency, mobile lifestyle, problem smartphone use, digital wellbeing

## Abstract

**Background:** Problematic smartphone use is widespread, and college-age youth faces an especially high risk of its associated consequences. While a promising body of research has emerged in recent years in this area, the domination of quantitative inquiries can be fruitfully and conceptually complemented by perspectives informed through qualitative research. Toward that end, this study aimed to interrogate the myriad behavioral, attitudinal, and psychological tendencies as a side effect of college students’ engagement with the smartphone in their everyday lived experience through in-depth interviews.

**Methods:** We recruited 70 participants from seven college campuses hailing from different geographic regions in China, and conducted semi-structured in-depth virtual interviews via WeChat in November and December 2020. Subjective experiences, personal narratives and individual perceptions in the context of routine interaction with the smartphone were thematically analyzed through a reiterative process in an effort to detect prevailing threads and recurring subthemes.

**Results:** The smartphone has established a pervasive presence in college students’ everyday life. Time-based use characteristics generated a typology of four distinct user groups: hypo-connected antagonists, balanced majority, hyper-connected enthusiasts, and indulgent zealots. Habitual usage falls on predictable patterns matched onto temporal, locale-based and contextual cues and triggers. Students’ dependency relationships with the smartphone have both functional and emotional dimensions, as prominently manifested in occasions of detachment from the device. Self-regulatory effort in monitoring and limiting use is significantly impacted by mental focus and personal goal setting. Perspectives from our qualitative data suggest the need for taking into account a variety of contextual cues and situational factors in dissecting psychological and emotional outcomes of smartphone use and abuse.

## Introduction

The rapid and widespread penetration of mobile technologies into the fabric of everyday life has fundamentally changed the landscape of human communication. This mobile revolution has been amplified by two landmark developments in the 21st century: mobile phone subscription surpassed fixed-line use in 2002 (Srivastava, 2005), and Apple launched its first iPhone in 2007 (followed by Google’s Android devices in 2008). By incorporating multifunctional applications and multifaceted traits into an all-in-one device, smartphones have nourished “an [sic] historical movement toward a personal communication society” (Campbell and Park, 2008, p. 381). Thanks to their boundary-spanning nature, portable convenience and all-encompassing affordances, smartphones function as integrated environments of polymedia (Madianou, 2014), and have turned into the “fourth screen” (coming after but emulating the historical role of the cinema, television, and computers) (Miller, 2014).

The pervasiveness of mobile media in general and smartphones in particular with the adolescent population is a hallmark of contemporary youth culture. As “mobile natives,” Vanden Abeele (2016) argues that immersive engagement with the smartphone has engendered heterogeneous “mobile lifestyles” among the current youth generation. Smartphone technology conforms to the *Apparatgeist* of “perpetual contact” – “the spirit of the machine that influences both the design of the technology as well as the initial and subsequent significance accorded them” (Katz and Aakhus, 2002, p. 305). The always-on mode of the smartphone, coupled by its portability and multilayered functionality, has triggered concerns about its addictive potential, especially among the adolescent and youth population. Against this backdrop, an expanding body of academic inquiries in recent years has linked excessive smartphone use to a variety of addiction-like behavioral and psychometric symptoms such as decrease in productivity and daily interruptions (Duke and Montag, 2017), stress, social anxiety and loneliness (Vahedi and Saiphoo, 2018), neuroticism and impulsivity (Carvalho et al., 2018).

The term “smartphone addiction” has been prevalently used and frequently studied in conceptual frameworks commonly adopted for substance abuse and pathological gambling in contextualizing its antecedents as well as myriad negative physical and physiological outcomes and consequences (Mahapatra, 2019; Sahu et al., 2019; Yu and Sussman, 2020). However, we concur with Panova and Carbonell (2018) that, even though there is mounting evidence to associate smartphone use with various problems and negative outcomes, “addiction” is not an accurate or correct term to diagnose a set of psychological or physical consequences that are not comparable to the severity and/or associated health problems caused by substance addiction. In a similar vein, as De-Sola Gutiérrez et al. (2016) point out, the diversity of perspectives encapsulated in the umbrella term and the failure to differentiate between addiction, problematic use and abuse has caused confusion and muddied comparability of findings.

It is worth noting that different terms – among them smartphone addiction, smartphone use disorder, pathological smartphone use, excessive smartphone use, maladaptive smartphone use, smartphone dependence, and problematic smartphone use – have been used interchangeably or synonymously in most academic literature. We support the call (De-Sola Gutiérrez et al., 2016; Panova and Carbonell, 2018) for a more precise conceptualization of terms, which is more constructive in promoting academic deliberations in investigating symptoms and pondering corresponding corrective actions. We therefore adopt the term problematic smartphone use (PSU) in our research, which aims to examine the myriad behavioral, attitudinal, and psychological tendencies as a side effect of college students’ engagement with the smartphone in their lived experience. We resorted to semi-structured in-depth interviews with 70 college students in disentangling the variety of nuanced pathological habitual patterns and psychological predispositions in the context of students’ daily interaction with the smartphone.

### Problematic Use of the Smartphone: Psychological and Behavioral Dimensions

By consolidating computing, portability, and mobility into one interface, the smartphone has the potential to fulfill a variety of communication needs from information to entertainment and interaction. The all-in-one nature of smartphone technologies has drastically enhanced the ever-expanding repertoire of available functionalities and applications. However, availability of services is not tantamount to adoption by the end users. As is the case with most other media technologies, usage and adoption of mobile applications and services has been a well-trodden area of academic research in the new millennium (e.g., Park and Chen, 2007; Verkasalo et al., 2010; Kang and Jung, 2014). The continuous advancement of smartphone technologies calls for constant update of this line of research in various national contexts.

*Research Question 1: What are the most frequently used smartphone-based apps in college students’ daily routine engagement?*

Design of the smartphone succeeds on a variety of habit-forming technologies and compulsive human tendencies (Eyal, 2014). As a result, habitual use of the smartphone has the potential to develop into certain patterns of compulsive behaviors, including repetitive checking (brief sessions of touching), context-dependent triggered acts, and quick access of dynamic content, all of which may induce habit formation on users (Oulasvirta et al., 2012). Psychology of habit theory posits that a variety of cues, exposure to which may be intentional or inadvertent, can trigger habit performance; in the case of substance use, addiction results when motivation shifts from goal-directed (voluntary) to habitual drug use (Wood and Rünger, 2016). It stands to reason that the same process applies to pathological smartphone use, although more research is needed in support of this mechanism. We therefore pose the following research question:

*Research Question 2: What are the temporal and venue-based cues and triggers driving patterns of habitual use of the smartphone?*

In terms of psychological consequences, a meta-analysis of 30 independent samples by Vahedi and Saiphoo (2018) confirms a positive association between PSU and stress and anxiety. A survey of college students in Turkey by Enez Darcin et al. (2016) found that social anxiety and feeling of loneliness are associated with vulnerability to smartphone addiction. In a similar vein, Yang et al.’s (2020) meta-analytic review of 14 studies points to a significant correlation of PSU with poor sleep quality, depression, and anxiety. Recent research has started to pay attention to NOMOPHOBIA or NO MObile phone PHOBIA, which is a psychological condition caused by the mental disorder over fear of being disconnected from the smartphone (Yildirim and Correia, 2015; Bhattacharya et al., 2019). Another behavioral tendency, especially among adolescent and young users, is called “phubbing,” defined as the practice of “an individual halting face-to-face communication with another person to interact with their telephone” (Erzen et al., 2021, p. 57). Moreover, problematic smartphone behavior can be exacerbated by FOMO, or Fear of Missing Out – the perceived need to be constantly connected over the apprehension of missing important information, especially that over social networking sites (Wolniewicz et al., 2018; Elhai et al., 2020). We are thus interested in finding out:

*Research Question 3: What are the college students’ self-reported symptoms and motivating factors with regard to NOMOPHOBIA, Phubbing and FOMO?*

There is a growing awareness among the general public about the excessive amount of time the smartphone consumes and its possible negative consequences on personal health and well-being. In response to the concerns of deepening dependency on the smartphones, digital detox has been proposed as one viable solution to promote planned abstinence from electronic devices such as the smartphone. A synthesis of existing evidence from the body of detox scholarship published between 2008 and 2020 as it relates to smartphone use shows mixed results, with no consistent findings between detox interventions and subsequent cognitive and physical performance measures (Radtke et al., 2021). We would like to contribute to this emerging line of research by asking:

*Research Question 4a: What detox measures, if any, do students undertake to mitigate smartphone (over)use?*

*Research Question 4b: What is the efficacy of these detox interventions?*

It is worthy to highlight that the majority of the research on smartphone use and addiction has been inspired by quantitative studies. For instance, an extensive review of current research on phubbing by Al-Saggaf and O’Donnell (2019) led them to bemoan the paucity of qualitative studies and prompted them to call for more qualitative interviews in offering rich descriptions on why people phub. What we aim to contribute to the expanding body of literature through our qualitative semi-structured interviews is to supplement and complement the sizable body of quantitative findings with in-depth, personal and situated perspectives to the diverse dimensions of PSU.

## Materials and Methods

We recruited university students from seven college campuses in China, hailing from different geographic locations and representing diverse academic disciplines. Interviewees were briefed on the overall purpose of the study as well as the voluntary nature of participation, and these who agreed to proceed were asked to sign an informed consent to take part in the study. Participants were assured of the anonymity of the interview data. We resorted to a semi-structured interview design in an effort to “understand themes of the lived daily world from the subjects’ own perspectives” (Brinkmann and Kvale, 2018, p. 14) with regard to their daily encounters with the smartphone. Because the interviews were conducted in November and December 2020 when the Covid-19 pandemic was still a threat, we adapted to a virtual interview modality conforming to the overall strategies and suggestions in Gray et al. (2020) and Khalil and Cowie (2020) from subject recruitment to rapport building to question handling and use of verbal/non-verbal cues, the main rationale of which was driven by concern for the participants’ health and well-being. However, one major difference is that, instead of using Zoom or Skye, we adopted WeChat, the most popular real-time chat app in China, to conduct the interviews. The reason is that all students have a high level of familiarity and comfort with video-chatting on WeChat, a routine engagement in their daily communication. Our overall WeChat interview experience corroborates the observation by Jenner and Myers (2019), who conclude after comparing Skye and in-person interviews that virtual interviews are conducive to more sharing of personal information, and does not compromise rapport or reduce the efficacy of the interview methods. As a result, we did not sense any loss or inferiority of the data thus obtained.

The questions cover a range of activities and user characteristics, with most of them open-ended in nature so as to capture the nuanced variations and diverse meanings each interviewee might assign while describing their everyday engagement, but all questions maintained a focus on themes pertaining to the various aspects of PSU mentioned in the above literature review. Specifically, we developed a few clusters of questions focusing on topics framed in our research questions, such as most-often used apps (RQ1), patterns of habitual use and responses to situated cues (RQ2), symptoms of NOMOPHOBIA, phubbing behavioral tendencies, how they would respond to leaving their smartphones behind, and FOMO (RQ3), and whether they had taken detox measures (RQ4a), and (if yes) to what effect (RQ4b). Each interview typically took 30 to 40 min to complete, with a few having gone more than 1 h. Follow-up questions were asked whenever necessary for the sake of clarification or data enhancement.

Data were analyzed by following the well-established qualitative content analysis and thematic analysis approaches in dissecting manifest content into categories and latent content into thematic threads (Guest et al., 2011; Vaismoradi et al., 2016). Our analysis is also inspired by the grounded theory method through immersing ourselves in the data corpus in pinpointing key concepts via microanalysis of specific topical areas as well as identifying salient patterns and thematic threads at the level of general analysis (Brinkmann and Kvale, 2018). Conforming to the often-adopted practice of processing the data through a reiterative process in analyzing qualitative interview data, we went through multiple rounds of analysis in first detecting core discrete concepts at the local/individual level and then deciphering dominant, tacit thematic alignments regarding broad perspectives from integrating the totality of the data.

## Results

### Interviewee Demographics and User Typology

The interviewees comprised 52 female and 18 male students. The disproportionate male/female makeup largely reflects the distribution of the gender differences in the disciplines in the host universities, which are dominated by humanities, social and management sciences, although the number is slightly skewed to the female line.

Of necessity, smartphone dependency first manifests itself in the amount of time one engages with the device on a daily basis. We asked each interviewee to offer an estimate on how much time the smartphone consumes them every day by turning on the Screen Time feature on their smartphone. The majority of the students were able to offer pretty precise answers, typically to the hour with some indicating a clear range (e.g., 6–7 h); moreover, about a quarter of the students reported the exact time as revealed on the Screen Time, such as 5 h 36 min on weekdays by one student. Based on their estimations, the average number of hours on the smartphone approximates to about 6 h (5.7 vs. 5.9 along male/female line) per day on weekdays. There is a sizable increase to the weekend hours (7.7 for male and 7.9 for female). There is, however, significant variation among the individuals, as the reported daily smartphone hours ranged from just 50 min to 10 h on an average weekday, and from 20 min to 12 h on weekend days. For the vast majority of students, there is a consistent pattern in the weekday-weekend variation; we therefore arrived at the daily average use amount in terms of hours by adopting the respective mean of weekday and weekend hours for each interviewee. Our tabulation of users in conformity to the daily amount of smartphone engagement yields four types of users, as reported in Table 1: below average (disciplined) users (less than 4 h on the phone per day); average (balanced) users (spending 4–7 h on the phone per day); above average (heavy) users (being on the phone for 8–9 h every day); and excessive (problematic) users (using 10 h or more for smartphone-related activities).

**TABLE 1:** Typology of users.

Gender	Below average (disciplined user) less than 4 h		Average (balanced majority) 4–7 h		Above average (heavy user) 8-9 h		Excessive (problematic user) 10 h and above		Total
Male	1	5.6%	10	55.6%	4	22.2%	3	16.7%	18
Female	3	5.7%	26	50.0%	17	32.7%	6	11.5%	52
Male and Female	4	5.7%	36	51.4%	21	30.0%	9	12.9%	70

*Total percentage may not add up to 100% for some columns due to rounding errors.*

Among the four individuals (three in the female group and one from the male group) who said they spent less than 4 h on an average day, all indicated the exercise of self-imposed control as an intentional effort to reduce the amount of time on the phone. On the opposing end, the nine students (making up about 12.9% of the total) who reported excessive smartphone use consented to symptoms of problematic or pathological dependency by explicitly admitting an urge to get onto the smartphone whenever possible. The following quote illustrates the all-consuming nature well by one interviewee: “I get onto my phone whenever I am free. Especially when it comes to the weekend, I stay on my phone all the time except for eating meals or taking the bath.”

For ease of cross-type comparison, we summarized time-based pattern of smartphone use in association with psycho-attitudinal responses to the interview questions into four distinct groups, as presented in Table 2. First, the *hypo-connected antagonists* (5.7% of the total) recognize the utilitarian aspects of the smartphone, and their engagement is driven by a highly goal-oriented approach in that they mostly know what they are looking for and go directly to the respective app, dominated by informational and social networking needs, accomplished in short sessions. They are also quite cautious about the negative potentials of the smartphone and exercise appropriate self-control. Second, the *balanced majority* (51.4%) maintain a conspicuous presence on the smartphone by spending 4–7 h on it. Their use is more expansive, as a significant amount of time is consumed in activities such as listening to music, video-sharing, and mobile shopping beyond information-seeking and social networking. They typically spend half to 1 h browsing the phone before bed and display more noticeable tendencies than hypo-connected antagonists symptomatic of NOMOPHOBIA, phubbing, and FOMO.

**TABLE 2:** Psycho-attitudinal traits of different user types.

User type responses	Below average users (hypo-connected antagonists)	Average users (balanced majority)	Heavy users (hyper-connected enthusiasts)	Excessive users (Indulgent zealots)
Most used apps	Social networking; news and informational browsing	Social networking; news and informational browsing; video-sharing services; music; shopping	Social networking; news and informational browsing; video-sharing services; music; shopping; entertainment	Social networking; news and informational browsing; video-sharing services; music; shopping; entertainment; gaming; live streaming
Bedtime and Wake-up Use	20–30 min before bed; 5–20 min after wake-up	30-60 min before bed; 10-20 min after wake-up	0.5-1.5 h before bed; 10-20 min after wake-up	0.5 – 2 h before bed; 10-30 min after wake-up
Phone out of sight	Feel calm (50%); Lose sense of safety (50%)	Feel uneasy, anxious or unsafe (73%);	Feel disconnected, insecure Cannot concentrate; Don’t feel myself (85%)	Feel anxious, a sense of loss, agitated (100%)
Think about the smartphone during class	Sometimes (25%)	Often or sometimes (52.8%)	Often or sometimes (76.2%)	Often or sometimes (77.8%)
Phubbing	Occasionally (25%)	Sometimes or often (51%)	Sometimes or often (75%)	Sometimes or often (78%)
When leaving smartphone behind	Feel unsafe (50%); Feel just fine or even happy (50%); Must go back and get it (25%)	Feel agitated, unsafe, antsy, bored (81%); Must return and find it (65%)	Feel edgy, bored, unfocused, isolated (95%); Must go back and get it back (45%)	Feel uneasy, disconnected from the world (89%); Must go back to pick it up before doing anything else (89%)
Worry about smartphone time and tried to reduce	Yes (25%)	Yes (51.4%)	Yes (38.1%)	Yes (77.8%)

The third user type, which we name *hyper-connected enthusiasts*, comprises 30% (22.2% male vs. 32.7% female) of the participants. Hyper is indicated by the level of smartphone engagement as measured in the amount of smartphone time (averaging 8–9 h per day), and enthusiasm is embodied in the palpable craving we detected in their interview conversations while discussing smartphone activities as well as their related emotional dispositions therein. Compared with the two previous groups, entertainment use (e.g., watching teledramas, reading online fiction, viewing movies and using TikTok) is an important part of their regular engagement with the smartphone.

The fourth cohort – who we call *indulgent zealots* – spend almost all their time outside of class and free from other required duties on the smartphone (averaging about 10 h per day). Although amount of time alone should not be the sole criterion, it is one of the most dependable benchmarks in diagnosing PSU in extant research (Duke and Montag, 2017; Vahedi and Saiphoo, 2018; Sahu et al., 2019). The statistical distribution of this group (12.9%) fits well-nigh the overall estimate by Eichenberg et al. (2021) in evaluating the prevalence rate of PSU at 15.1% in their study of college students in Vienna. Besides the prevailing tendency to stay longer on a variety of activities that the previous groups also engage in, close to one-half of them specifically mention mobile gaming as one of the most frequently accessed apps on their smartphone. Of particular note is that these students consistently display a set of psych-behavioral traits commonly associated with PSU, such as NOMOPHOBIA, FOMO, and worrying about the amount of time consumed by the smartphone.

The fact that gaming has been mentioned the most prominently among the indulgent zealots is noteworthy, as gaming has been consistently pinpointed as a primary addictive tendency associated with compulsive smartphone use (Liu et al., 2016; Derevensky et al., 2019). However, PSU symptoms are not just limited to indulgent zealots only, as similar patterns (albeit to a slightly lesser extent) can be observed with hyper-connected enthusiasts. With regard to content type, the reported use pattern among our cohorts is highly congruent with research findings linking entertainment use and gaming to problematic smartphone dependency (Jeong et al., 2016; Bae, 2017; Park et al., 2021).

The overall patterns of differences across the four user categories can be found in Table 2. Detailed symptomatic manifestations among the interviewees are discussed in the sections that follow along the topical lines of the research questions.

### Smartphone Utilities (RQ1)

We asked each participant to name five to six apps that they used the most frequently. Among the most mentioned are a total of about 20 apps encompassing four broad areas of functions and affordances. Ranked in the degree of their popularity, the first category serves to carry out variegated tasks of socializing functions via instant video and text messaging, as seen in WeChat, QQ, and Sina Weibo. The second type of apps pertains to multiple ways of news sharing and information seeking (e.g., WeChat, QQ, Toutiao, Zhihu). Closely aligned with the second type is an assortment of apps – for example, Xiaohongshu, Taobao, Alipay, Elema – that facilitate the delivery of utilitarian transactions and tasks ranging from online shopping, mobile payment, photo-taking, and time-keeping to navigational services. Trailing not far behind, the fourth category of apps cater to students’ entertainment needs, as exemplified by NetEase Cloud Music, QQ Music, blibli, Youku, and mobile games led by *King of Glory* (also known as *Wangzhe Rongyao* in Chinese) and *Counter-Strike*.

Table 3 lists the top 10 apps students reported using the most. Of particular note is the role of WeChat in the routines of everyday communications among the participants. WeChat offers multifunctionalities that crosscut boundaries typically found in the first and second types of apps as mentioned above – its text messaging, audio and video chat features are widely used for one-on-one interpersonal communications, while the group chat and one-to-many broadcast capabilities make it the platform of choice for getting messages out to groups of varying sizes. The latter affordances make WeChat a hugely popular venue for information sharing, as evidenced in the avowed use of WeChat by the students as a major channel of information seeking.

**TABLE 3:** Top 10 most often-used smartphone apps.

Rank	App
1	WeChat
2	QQ
3	Alipay
4	NetEase Cloud Music
5	Taobao
6	Sina Weibo
7	Eleme
8	Baidu Cloud
9	Bilibili
10	Xiaohongshu

Along gender lines, we noted a striking difference: male students express an unmistakable appetite for games, whereas female students are much more inclined to use the built-in camera. *King of Glory* dominates mobile gameplay. Conversely, almost all female students admitted to using the built-in camera as one of their favorite habitual undertakings while only about half male students acknowledged doing this. The most common cited motive for photo-taking is to chronicle daily life, as revealed in this quote: “Shooting pictures is my favorite pastime. I send photos of my every meal to my parents. I will take photos of the scenery or objects I like whenever I take a stroll.”

One notable development in China during the past years has been its quick transformation into a cashless environment enabled by the widespread adoption of mobile payment technology. This is resonated soundly in the interviews, and it comes as no surprise that e-payment via the smartphone is one of the most sought-after features among the students. This is well illustrated by the following statement:

“One cannot be separated from the smartphone nowadays, mainly because it fulfills the daily need of paying bills. Like most everyone else, I used to pocket cash a few years ago. Now that’s no longer necessary thanks to the smartphone. I can almost do any transaction with the phone, such as buying stuff, paying fees and purchasing tickets. So the smartphone is indeed all-capable!”

Indeed, many students specifically mention e-wallet as one of the major causes of their anxiety when asked about how they feel when they don’t have their smartphone with them. This is elaborated in the ensuing discussion on NOMOPHOBIA.

### Habituation (RQ2)

As mentioned in the previous literature review, checking behaviors comprise a large part of repetitive habitual use of the smartphone (Oulasvirta et al., 2012), and smartphone-related habits are closely associated with external situations or internal states (Wood and Rünger, 2016; Park et al., 2021). Research also indicates that process-oriented smartphone use may develop into habits, which may in turn automatically trigger problem behaviors activated by internal or external cues (Van Deursen et al., 2015). We asked the students about their habitual routines, rituals, and general tendencies in using their smartphones. The following thematic lines stand out across the interviewees.

A clear pattern emerges characterizing students’ interaction with the smartphone: almost all indicated that the time they spent on the phone surges during weekend or holidays; and smartphone is the primary medium of choice when fragments of time are available, such as during intervals between routine tasks, and moments of non-essential or leisure activities. Smartphones are the unrivaled choice for casual browsing, as students typically opt for “swiping” at most chunks of time available. As far as gender is concerned, female students display no significant deviation percentagewise in these behavioral patterns.

The venue that smartphone consumes the most uninterrupted chunk of time is the dorm for most students. The most extensive block of concentrated smartphone time on each day for virtually everyone is the pre-bedtime hours, although the specific amount of time varies from about half an hour to more than 2 h. The next routinized allocation of smartphone time prevalent among the students is the early morning hour, when the students typically idle on bed from 15 min to close to 1 h browsing the smartphone. Besides the difference in length of time, the late-evening and early-morning rituals tend to focus on different tasks and accomplish different purposes. Late-evening smartphone use is primarily entertainment-oriented (e.g., video, online teledramas, music, gossipy tabloid hearsays, gaming), although socializing (e.g., personal communication) maintains a noticeable presence. Early-morning smartphone checking uniformly centers on updating news of the day and attending to personal messages. As result, students mentioned gravitation toward different apps during these two daily periods. It is also worthy of note that the pre-bed period shows a distinct pattern of variation among the different types of users (from below average to excessive users) in the amount of time they expense. Especially among heavy and excessive users, many confess that this has become a basic routine as a necessary precursor to sleep every night. In contrast, the early-morning time immediately after wake-up, which is typically followed with some smartphone browsing, does not vary much with user types, with each student spending anywhere between 10 and 20 min doing this. This is understandable in that morning is not the time for most students to loaf around in bed, as they are rushed to get ready to embark on the errands of the day.

Besides the pre-bed hour, the next block of time of concentrated smartphone use for the students is during meal (i.e., lunch and dinner) time, when casual, entertainment use dominates. This is confirmed both from self-revealed narratives and alleged observations of habitual behaviors by others. This behavior is aided by the design of the smartphone for one-handed holding and swiping, as some students acknowledged. Another favorite way for the students to engage is to place the smartphone on the table and browse content on the smartphone while dining. Ease of single-handed actions such as flicking, tapping and dragging is something that many students fondly describe and have become very adept in doing.

Regarding the question whether they turn their smartphones off while going to sleep at night, only two out of the 70 informants answered positively, while the rest confirmed that they always keep their phone on at night. Of the two who turned their smartphones off, one student indicated doing this as a habit formed years ago, and the other student who turns off the phone at night said she does this due to health concerns:

“I used to turn the phone to airplane mode, but I was told that would not totally eliminate radiation [from the phone]. In order to avoid radiation, I now completely switch the phone off.”

As to why they keep their smartphones on at night, the most-cited reason (by 83% of the students) is to use its alarm and time-keeping function. Ninety percent of the interviewees said they placed the phone within grab distance, while about a quarter of the students mentioned checking the time on the smartphone at night. Psychologically, about a quarter made a point that opening eyes to see the smartphone makes them feel safe.

In response to the question whether they would check on the phone while waking up in the middle of the night, 32 (constituting 45.7% of the total) students admitted doing this. The type of content consumed late into the night varies quite a bit from looking at the current time, checking on friends’ WeChat “Moments” posts and Weibo updates to viewing short videos. When asked whether the late-night smartphone feeds had any negative impact on their sleep quality, 44% said no but 56% answered in affirmation. Similarly, the aftereffect of this midnight smartphone perusing is diametrically perceived by the two groups: the former group claimed that doing this helps sooth them back to sleep whereas the latter group alleged smartphone checking during bed hours often produces some type of arousal effect on them, thus prolonging the time they need to go back to sleep. Some students in the latter group, albeit not specifically acceding to being addicted to the smartphone, alluded to the potential nature as shown in these quotes:

“Checking my smartphone at night affects my sleep. Oftentimes, once flipping the screen on, it keeps me in a state of arousal, and delays my time to go back to sleep.”

“If I get onto sites such as Zhihu [a popular question-and-answer site like Quora] and Weibo, my sleep will suffer, because these sites are highly addictive. Related content through links on these pages is very seductive to thoughtless, mechanical strolling.”

College students’ social life mainly consists of moments such as hanging up with peers during class breaks, meal times, or weekend hours. Smartphone has been invariably cited as the most-sought-for companion for various purposes – kill time, fool around, idle away, or finish fragments of academic assignments. The campus lifestyle dictates a lot of in-transit moments when students move around between the dorm, classroom, cafeteria, and other places in attending to daily tasks and events. Listening to music is a popular activity for these students, as well as some occasional “virtual strolling” into quick informational checking via various apps of personal preferences. Fifty-six percent of the students acknowledged that they are in the habit of checking their smartphones on a regular basis while walking. As corroborating evidence for its popularity, in response to our interview question on what the students saw as the most common behavioral habits among their peers, topping the list was smartphone checking while walking, followed by looking at the smartphone during meal time and holding the smartphone in the hands at all times.

The habit-inducing nature of the variety of features in the design of the smartphone and its apps is duly noted by the students. Many students explicitly pointed out that they are sensitive to all sorts of prompts and hints (e.g., tones, vibrations, flashing signals) from the phone, and have developed a compulsion to check it out, even if this is during class or late at night. Some students conceded to the irresistibility to upgrade at seeing the little red dot reminder that all brands of smartphone products have adopted indicating availability of newer versions of apps or latest system upgrades. Moreover, AI-operated apps to customize content to individual users are particularly powerful in getting users “hooked.” One student expressed both her fascination and trepidation about Zhihu, a Quora type of peer-to-peer Question-and-Answer app this way:

“At the start, I feel at total control. But the more I click on the app, the more I am trapped into it. In the blink of an eye, 20 min or more has flown by without me knowing it. I may feel it is a total waste of my time doing this. But next time I repeat doing the same thing [on the app].”

### NOMOPHOBIA, FOMO, and Phubbing (RQ3)

An emerging line of research in recent years has ascertained the association of nomophobia with a number of negative outcomes pertinent to fear, stress, panic, and anxiety due to inability to access the smartphone (aka nomophobia) (Nie et al., 2020; Rodríguez-García et al., 2020). College students suffering from symptoms of nomophobia tend to struggle with concentrating in class (Lee et al., 2017) and perform poorly in academic achievement (Gutiérrez-Puertas et al., 2019). In order to contribute to this body of research, we asked questions of interviewees as regards the degree of pervasiveness of nomophobia and its varied symptomatic manifestations through a set of questions about their attitudes and personal experiences of dealing with situations absent of the smartphone. One question pertains to whether they think of their smartphones during class hours. Forty-three (or 61.4%) of the 70 students interviewed said they often or occasionally get distracted by thinking of their phones, with about 43% acknowledging occasionally engaging in quick phone checking during class. The reasons mentioned for the distraction are mostly one of the three (ranked in this order): the class gets uninspiring; there is an anticipation of time-sensitive information; and there is no specific reason other than the phone just pops up in the mind. Another question asked them if it is their habit to regularly check their smartphones while engaging in tasks such as academic homework, reading and exercising. While about half of the students said they can stay focused on these activities, 37.1% admitted to frequent phone checking while doing these things. It should be noted that the lattermost category involves not merely a quick thumbing through or transitory swipe of the smartphone; this rather entails extensive, concomitant use in parallel with other activities.

In response to the question how they feel when the phone is out of sight, approximately 18.6% (*n* = 13) said they would stay calm and cool-headed, vis-à-vis the rest of the 81.4% expressing varying levels of anxiety ranging from feeling insecure to panicking and agitation. As summarized in Table 2, the most common answers are feeling unsafe, disconnected, uneasy, anxious, a sense of loss, and agitated, whose level of severity steadily increases in accordance with the scale of smartphone dependency in the four user groups. Reversely, the percentage of calm-minded students while the phone is out of sight shows a counter trend – 50% for the below-average group, 22.2% for the average-use group, 14.3% for the heavy-use group, and 0% for the excessive-use group. The pattern along level of smartphone use versus frequency of phubbing and phone checking in the middle of the night (see Table 2 for details).

Relatedly, we asked the students whether they had left their smartphones behind when going out for the day in the recent past, and if yes, what they had done. Twenty-seven students answered firmly that they had not left their phone back, and what is striking are the reasons they cited for *why this had not happened* – the consistent line therein is that the smartphone constitutes such an all-pervasive aspect of their everyday life that it is virtually impossible to go out without the phone. This sentiment is typified in these two remarks:

“I won’t forget my smartphone any day, because whenever I walk out of my dorm, the first thing I look at is my phone. I wouldn’t walk further beyond a few steps on the stairs before I found out that the phone was not with me.”

“The smartphone is more than just a device of communication; it is a part of my body organs. The moment it’s not with me, I will immediately notice. So I won’t go out without my smartphone.”

Of the 43 students who had experience of leaving their smartphones behind, the words that the students used to describe their feelings at that moment are (ranked in frequency): panicking, uneasy, distressful, restless, unsafe, scared, at a loss, detached from the world, bored, strange, and in despair. Interestingly, the key words mentioned by our interviewees bear substantial semblance to those used by undergraduate students in Furst and Evans’s (2021) campus intercept interviews on students’ reactions to temporary loss of possession of the smartphone. On the other end, only two students said they were “feelingless” (emotionless or unmoved). This response is quite typical:

“I remember one time I did not have my smartphone with me. Without it, I didn’t have any sense of safety, and felt very isolated, to the point of despair. The whole world felt strange to me, and I didn’t know what to do.”

As to what they would do next, 26 (60.5%) said adamantly that they must find a way to immediately go back and retrieve the phone, because otherwise they would not know how to make it through the day. Eleven said that they would wait a bit until they finished what was at hand and then find an opportune time to go back and fetch the phone. A common sentiment among these students during the time without the smartphone was that the time passed by unbearably slow, and they felt “strange” and “out of place” while seeing others were on their smartphones. Only six indicated that they could sustain the day without the smartphone, albeit not without any difficulty for everyone. Being away from the smartphone brought about some unanticipated jubilation for a few:

“I initially panicked a bit [being away from the phone]. But after a while, I actually started to feel relieved at the thought of spending the way without the smartphone. It gave me a sense of comfort that this would be a day without the [virtual] crowd, free from messages and updates, a day when I could relax.”

“It felt weird at the beginning. But I got over that quickly, and gained a sense of elation [at not using the smartphone for the day]. I was able to focus my attention on other things and made a good day of it.”

The haptic benefits, portability and personal nature of the smartphone may cultivate relationships beyond its practical and functional use, as users may “experience enhanced psychological comfort from engaging with their device, which allows it to serve as a palliative aid for owners during moments of stress” (Melumad and Pham, 2020, p. 251). Over 60% of the interviewees expressed a psychological sentiment of comfort and reassurance while physically holding the smartphone in their hands. The absence of the smartphone from their sight, or an extended period of time (which typically lasts a few minutes for most students) of not checking the phone creates a particular type of anxiety or distress triggered by FOMO among 68.6% of the students. Specific behavioral responses to mitigate FOMO cited by the students vary from constantly keeping an eye on the phone for cues (e.g., audio alert, vibrating notifications, customized prompts) to frequent phone checking to getting up at night hours for an quick updated skimming.

When asked if they would check the smartphone instead of paying attention to their companions during social conversations (phubbing), forty (constituting about 57%) out of the 70 students admitted doing this often or sometimes. The most-cited reasons for opting to do this are (ranked from high to low): to bypass boring conversations; to evade awkward moments with people they do not know well; not to miss important smartphone messages from friends; and others are looking at the smartphone. Close to 30% of the students mentioned phubbing as a social strategy during moments when they do not have anything to say or when they want to avoid speaking, especially in the company of others they do not perceive as intimate friends. Ten percent of the students alleged that they can manage to multitask between conversing with friends and checking the smartphone without affecting either in any negative manner. As a matter of fact, the prevalence of phubbing-related behavior in China in recent years has even led to the coinage of a new word in the Chinese language – *ditouzu*, or the “Heads-down Generation,” to (derisively) refer to the tendency of people in late teens and early 20s to lower their heads in fixedly staring at the smartphone in social situations or while walking in public spaces.

Phubbing points to the increasing susceptibility of individuals to spend more and more time with their smartphones while less and less time engaging with each other, and may cause feelings of social exclusion, degrade interpersonal relationships, and impair personal well-being (David and Roberts, 2017). Responses to our question about the impact of the smartphone on interpersonal relationships are varied and can be thematically classified into four categories. About 45.7% (*n* = 32) of the informants answered in the affirmative (i.e., strengthening), because the smartphone has increased both the level of contact and the amount of content they exchange with their loved ones and friends. Many students stressed the affordance of the smartphone to enable constant engagement with their family even though they are separated from one another (living away from their families). On the opposing end, 30% of the interviewees felt that smartphone use has distanced them from their intimate circles, largely thanks to the reduction of face-to-face communications. An often-mentioned scenario is the decrease of conversations among family members while being together, and a few students admitted that the overreliance on the smartphone has impaired their competence to relate to their loved ones. About 12.9% (*n* = 9) of them said the smartphone has had no impact on their relationships with family and friends, while 11.4% reported mixed reactions (i.e., weaking some relationships but strengthening others).

### Detox and Self-Regulation (RQ4)

With regard to our inquiries on whether the students made any efforts in cutting or controlling the amount of time they spend on the smartphone, thirty-six indicated they were not concerned about their smartphone time, nor had they tried to curtail its use. Eight said they made sporadic attempts to reduce smartphone use, although they were not concerned about the amount of time they spend on the smartphone. Twenty-six (37.1% of the total) students expressed concerns over the amount of time they spend on the smartphone, and adopted measures in monitoring and reducing their screen time.

Among the 34 students who took effort to monitor and limit smartphone use, the most common way of doing this, as reported by 30 students, is to resort to popular apps such as FocusToDo, Plantie, Screen Time, Tomato Timer, Forest, TODO for managing time and forcing users out after extended use. Rate of digital detox app adoption varies substantially across the four groups of users we identified in Table 2: excessive and below-average users are diametrically disposed to adopt detox apps (66.7% vs. 25%), with average and above-average users in between (50% vs. 42.9%). Excessive users, who face the highest risk of problematic use, have the highest rate of adoption, which reflect the perceived need of this group in resorting to detox app in cutting down use. Additionally, we were interested in the effect of such apps on those who were intent on curtailing smartphone use, and therefore only asked follow-up questions of the 26 interviewees who explicitly professed such goals. Nineteen of the 26 agreed that their measures were effective while seven answered otherwise.

Using app is not the only means to exert self-regulation over smartphone use. What seems at play in the process is individual goal setting and mental focus, a repeated theme we observed across the interviewees. Many students pointed out that the smartphone only becomes the centerpiece of free play and the locus to idle away time when they are unoccupied or unengaged with anything else, or at moments they feel bored. They have therefore devised various strategies to steer themselves away from the smartphone by engaging in these activities as mentioned in the interviews: doing physical exercises, going on outdoor excursions, reading, chatting in person with friends, turning off the phone, or placing the phone away for the time being. In the case that non-smartphone activities are not an option, five students indicated that sleeping it out works to keep them unhooked from the phone.

Finally, although not a specific focus of our research, the role of the smartphone in the college learning environment has come up repeatedly in our interview conversations with the students. In China, like most elsewhere, more and more college campuses embrace the flipped classroom pedagogical approach, which is a learning model that subverts the traditional teacher-centered class instruction into student-focused pre-class knowledge transfer via technology-mediated platforms including smartphone capabilities (Wei et al., 2020). As a result, the smartphone has become an important and pivotal tool in fostering learning through entertaining, mobile gaming, and other creative modalities (Krouska et al., 2020; Troussas et al., 2020). More than one-third of the students mentioned the various role of the smartphone in accomplishing academic and course-related tasks such as researching information, communicating about curricular activities, and reading class notes and course materials. To some extent, the smartphone has assumed some functions that used to be fulfilled by personal computers in the college learning environment, as acknowledged in our interviews. In this regard, the amount of smartphone time will be skewed significantly for those who are more dependent on the smartphone for learning purposes, and type of activities, rather than smartphone time, should be a more reliable indicator of problematic use.

## Discussion

Our research set out to interrogate the multifaceted dimensions of PSU among college students in China. Informed by extensive data we gathered from semi-structured in-depth interviews of 70 undergraduate students from seven college campuses, our findings contribute to the expanding body of academic literature related to this area of research in several ways. First of all, the smartphone has established a pervasive presence and has become a defining feature of the everyday lifestyle among college students. The amount of time the smartphone consumes the students is staggering, averaging close to 6 h during weekdays and nearly 8 h during weekend days. Our typology of time-based smartphone use yields four distinct types of users: hypo-connected antagonists, balanced majority, hyper-connected enthusiasts, and indulgent zealots.

In studying employees’ experience with converged multi-functional mobile devices, Matusik and Mickel (2011) identified three types of users based on how they interpret and practice technology use: *enthusiastic reaction* puts a totally positive spin on the professional experience and perceives no cost; *balanced reaction* appreciates the benefits but also sees its downsides; and *trade-offs reaction* recognizes professional benefits but acknowledges significant personal costs with a common feeling of personal conflict and struggle in maintaining control. The smartphone use in our study differs from the previous context in that ours involves student users in a non-employment environment but the previous research includes mobile devices beyond the phone. Nonetheless, we found parallel as well as distinction between our groups and those by Matusik and Mickel. Our hyper-connected enthusiasts bear semblance to the technological enthusiasts as identified by Matusik and Mickel in that there is a noticeable craving for the smartphone among most of these students while discussing their smartphone use. The balanced majority revealed in our study share quite a bit with Matusik and Mickel’s balanced reaction group. Their trade-offs group is divided into two groups on the opposite end in our research, with the hypo-connected antagonists casting a cautious eye on the downside of the smartphone while the indulgent zealots totally embracing the technology in the other direction.

With respect to the utility aspects of the smartphones, it is easy to note that WeChat has taken supremacy as the all-in-one platform for social networking among Chinese users. Since the advent of short message services (SMS), text messaging and voice call have been two of the most prodigiously used features in mobile services (Ling, 2004; Karnowski and Jandura, 2014). Our research findings, however, suggest signs of seismic transformations in the smartphone era. Conventional voice calls, albeit still used on a regular basis by all the students, have become secondary in terms of the amount of time expended by most of them in comparison with other affordances available on the smartphones – so much so that voice calling does not even make it to the top ten of the features in consuming everyday time of the students. Text messaging has been sidelined even further, with just a few interviewees mentioning engaging in that occasionally. This is not to suggest, nonetheless, that these students have stayed away from voice communications or text messaging. Rather, the indications are that students have uniformly expressed preferences in embracing the built-in text, voice and video chat features with WeChat. There is a clear displacement effect in which conventional text messaging and voice call functions are migrating to alternative smartphone-enabled venues.

In its over 100 years of research, habitual use of technology has been consistently found to be moderated by mechanisms that automatically trigger repetitive behaviors in response to recurring context cues with varying (intermittent) rewarding outcomes (Bayer and Larose, 2018). It is important to note that habit automaticity is a necessary but not determining condition causing compulsive or addictive behaviors, as many other factors play an essential role in shaping the path to pathology (Wood and Rünger, 2016). Smartphone technologies give primacy to haptics (i.e., making touch an analog of seeing and hearing) (Parisi and Archer, 2017), a feature that is particularly malleable to the design and implantation of habit-forming interfaces and apps (Stawarz et al., 2015). Our research findings have imparted numerous temporal, locale-based and context-derived behavioral tendencies of smartphone use among the students.

Contextual cues and situational factors play a pivotal role in the formation of behavioral habits. Through an online survey, Karnowski and Jandura (2014) deduced three main mobile usage patterns – “Mobile@home” (among known peers in familiar locations); “En route” (on the way among unknown people in unfamiliar surroundings); and “Hanging out with peers” (with peers in unknown locations. Habitual practices are associated the most frequently with the residence (the equivalent of their “home”) by the informants in their interviews. Since all the students we interviewed are on-campus residents, the dorm is tantamount to the home of the employees investigated by Karnowski and Jandura, and their smartphone engagement bears some resemblance in that usage situations are the most dominant across interviewees. The “En route” moments for the students mostly comprise their in-transit time walking between the dorm, the cafeteria, classrooms and other venues on campus, while their “Hanging with peers” hours manifest profusely in the “empty” chunks of varying lengths such as intervals between classes and/or other obligated school activities, meal breaks and off-class hours. Our findings show that students’ smartphone usage has displayed predictable patterns in connection to these various occasions in terms of both app checking and content browsing. One word of caution, however, we should highlight is that same habitual predisposition should not be construed as unidirectional in its consequence. A case in point is smartphone checking during late night hours, which may work toward pacifying some students but arousing others, thus producing very different impact on their sleep quality. While a common finding in quantitative research suggests an association between PSU and poor sleep quality among adolescent and youth populations (Hale et al., 2019; Mac Cárthaigh et al., 2020; Yang et al., 2020), results in our interview suggest the need for taking into consideration contextual clues and situational factors in order to develop a more nuanced understanding in disentangling causational attributions.

It is probably no surprise that our research has lent evidence to the undisputable presence of widespread nomophobia and FOMO among college youth. This finds testimonial in various manifestations, from thinking about the phone during class, to keeping the phone in sight and within reach, to holding in hand and never having left it behind in the memorable past, the smartphone has assumed a role beyond that of a technical gadget in sustaining students’ emotional and functional stability. Results in our interviews indicate the level of smartphone dependency is positively related to the severity of disturbance while adversely related to the degree of self-imposture in a number of symptomatic manifestations under investigation.

Problematic smartphone use has emerged as an important public health issue in recent years, and both technical and non-technical interventions have been proposed as possible solutions to limit and control smartphone use (van Velthoven et al., 2018). The percentage of screen-time controlling app use in our cohort (42.9%) aligns up very nicely with Schmuck’s (2020) study, which found that 41.7% of the surveyed 500 Australian adults adopted detox apps to limit and control smartphone time. In addition, Schmuck alleges her research evidence shows “for the first time that self-monitoring behavior using digital detox apps may prevent young adults to develop problematic or compulsive smartphone usage patterns due to using SNSs” based on multigroup analysis that “those young adults who used digital detox apps indicated lower levels of perceived PSU and higher levels of well-being in response to the use of SNSs” (Schmuck, 2020, p. 26). Findings in our study, however, paint a different and more nuanced picture. That excessive or problematic smartphone users are the most likely to resort to detox apps in exerting self-control cannot be construed as evidence to either refute or confirm the efficacy of these apps or such a mechanism; it is plausible that excessive smartphone dependency tends to lead to self-monitoring through detox apps. Whether it is accomplished through technology-based detox apps or through non-technological approaches, we found strong evidence that mental focus and individual goal setting play a central role in the success or the lack thereof in outcomes of moderating smartphone use. This accords cogently with the core premise that the exercise of free will is “a *causal primary*” to effect self-regulation (Binswanger, 1991), and it highlights the critical role of self-monitoring and self-reaction as conceived in the theory of self-control (Bandura, 1991).

Lastly, the results of our research are best understood in the context of its limitations. Due to the qualitative nature of our research design, the number of students we studied, although more than sufficient for in-depth interviews, is a small sample size compared with large-scale quantitative studies. Correspondingly, the perspectives and insight we generated from the data may not be generalizable to the large population of college students in China. The findings we presented in the paper call for corroboration and triangulation from large-scale datasets derived from cross-sectional or even longitudinal surveys. Moreover, differences in national settings are likely contributors to variations in usage patterns; it is therefore useful to make cross-national comparisons in deepening our understanding of PSU among global youth.

## Conclusion

Problematic smartphone use is a pervasive phenomenon, and calls for attention from scholars with diverse backgrounds and contribution from multidisciplinary perspectives. Prevalence rate is particularly prominent among college-age population, as the smartphone has established itself as a hallmark of youth lifestyle. From its built-in technical features to the assortment of apps and the rich set of available content, the smartphone is conducive to repetitive, habit-forming patterns of usage. Students’ engagement with the smartphone often displays predictable behavioral proclivities in response to specific temporal, locale-based and contextually driven cues and triggers. While informational use is universally found among all users, problematic use is typically associated with gaming, streaming, entertainment, and social networking gratifications. As smartphone further establishes itself as a viable tool in mediating college learning, time alone should not be used as a sole predictor of problematic use. Both activity type and level of engagement warrant consideration in evaluating PSU. Extensive interaction with the smartphone has led to a special type of attachment to the device that pertains to not just its utilitarian functionalities but also its affective bond, manifested in various symptoms of uneasiness, discomfort and anguish at moments of not being with or seeing the smartphone. While we found evidence of the efficacy of detox apps in curtailing use, mental focus and proactive goal setting seem to be the most productive in attaining self-regulatory goals. Perspectives from our qualitative data suggest the need for a more nuanced approach in taking into consideration contextual cues and situational factors in dissecting psychological and emotional outcomes of smartphone use and abuse.

## Data Availability Statement

The raw data supporting the conclusions of this article will be made available by the authors, without undue reservation.

## Ethics Statement

The studies involving human participants were reviewed and approved by Institute of Scientific Research at Minjiang University. The patients/participants provided their written informed consent to participate in this study.

## Author Contributions

CD, ZT, and SN were involved in the conception and design of the study, coordinated work to transcribe, and analyzed the data. ZT wrote the draft of the manuscript. CD and SN arranged and conducted the online interviews. All authors contributed to the article and approved the submitted version.

## Conflict of Interest

The authors declare that the research was conducted in the absence of any commercial or financial relationships that could be construed as a potential conflict of interest.

## Publisher’s Note

All claims expressed in this article are solely those of the authors and do not necessarily represent those of their affiliated organizations, or those of the publisher, the editors and the reviewers. Any product that may be evaluated in this article, or claim that may be made by its manufacturer, is not guaranteed or endorsed by the publisher.
